# Summer Medical Schools

**Published:** 1850-04

**Authors:** 


### SUMMER MEDICAL SCHOOLS.

The regular lectures in these Institutions will commence on the first of April. A reference to the advertisement page, will exhibit a schedule of the courses in each. It may be stated, however, that not more than three lectures are delivered daily, and the hours are so arranged as not to interfere with those devoted to Hospital and Clinical instruction. By means of antiseptics, the lecturers on Anatomy are enabled to make their demonstrations from the recent subject, thus presenting the same advantages that are obtained during the winter, and with as little personal inconvenience.

